# On the Treatment of Bowel Affections of Infancy and Childhood, Incident to the Summer Months

**Published:** 1878-08

**Authors:** De Laskie Miller

**Affiliations:** Chicago


### ON THE TREATMENT OF THE BOWEL AFFECTIONS OF INFANCY AND CHILDHOOD, INCIDENT TO THE SUMMER MONTHS.

By De Laskie Miller, M. D., Chicago.

(*A Paper Read before the Chicago Medical Society, July* 15, 1878, *and published at their request.*)

Annually, as the warm season approaches, the attention of medical men, especially in large cities, is directed to the increasing prevalence of, and to the mortality from the bowel affections of children. So serious are these affections in unfavorable localities, that the ranks of this interesting class of our patients are decimated despite the most assiduous exertions for their relief.

The suggestions I may make this evening upon the treatment of the bowel affections of infancy and childhood, incident to the summer months, are presented in response to the request of your efficient secretary, who, I believe, should be credited largely for the prosperity of this society hitherto.

It cannot be claimed that my subject is new, nor would I lead you to expect that any element of novelty in its treatment will be advanced. It has been so frequently presented to this society that it may now well be styled trite. Wisdom and skill, however, come from ample facilities for observation extending over considerable time and space. I may well feel satisfied if the accumulated experience and wisdom of the members of this society present, shall be elicited, in a free discussion, which cannot fail to result in useful suggestions.

Would I present an intelligent résumé of the treatment of the bowel affections of infancy and childhood, I must enumerate some conditions which are predisposing, and some causes that are provocative. If some of these seem to be emphasized above that which is usual, it will be to direct especial attention, first, to prevention and, second, to restoration by the minimum of medication.

I am not unmindful that I am speaking to physicians as well qualified as I to appreciate the ever varying conditions present in these cases, and therefore shall not deem it necessary to elaborate the different topics in detail. The time at my disposal would not justify this.

Allusion in the briefest terms only, is all that will be required in regard to the anatomical peculiarities which predispose to abdominal disease in early life, such as the immature development characterized by softness of the tissues which exist in the mucous membrane its follicles and glands especially of the alimentary tract, and the peculiar arrangement of the stomach and intestines, which renders these organs more susceptible to irritants than in mature life. These are familiar to every physician, but are important as predisposing to vomiting, diarrhœa, gastro-intestinal inflammation, etc.

High temperature, by exalting nervous sensibility while tone is depressed, is doubtless an important factor in these cases. These effects of a high temperature upon the organism in early life, may be everywhere and always observed. They inhere, and must be accepted as inevitable. That high temperature alone is the cause of the high rate of infantile mortality does not appear tenable, else would the returns from country and city, more nearly correspond.

A vitiated state of the atmosphere is more potent in its deleterious effects upon the infant organization. This, too, must be accepted as inevitable in large cities. It is impossible to congregate half a million of people, and still enjoy a pure atmosphere. The consideration of drainage and ventilation comes not within the scope of this paper, however important they are conceded to be. In all cities, conditions exist which are deleterious to the public health, and cannot be avoided by any, except by migration. Causes of contamination, however, which by proper care might be obviated, exist in many dwellings. Medical men know too well, from daily personal experience, that the atmosphere of many dwellings, and these not always limited to the poorest classes, is unnecessarily vitiated to the last degree, by neglect of the most common attentions demanded in every household. Who among us, while making professional visits, has not detected repeatedly the sickening odors arising from decomposing excreta, which should have been promptly removed from the apartment, and fresh air admitted! It remains for the physician to suggest the changes, and to see that his commands are executed.

It will be proper here to consider briefly, a factor of greater importance in these cases, viz., the food suitable for infants. That furnished by nature, undoubtedly best fulfills the demands of infancy, and in the majority of cases leaves nothing to be desired. Still it does not follow that breast milk is always nutritious or even harmless. Every physician has frequently met with instances in which the milk of the mother, or wet nurse, acted upon the infant's stomach as a direct irritant. And this, too, when the mother or nurse appeared in excellent health. Milk to be acceptable should possess certain qualities, and be free from certain elements. It should be alkaline in its reaction. It is, however, so rapidly rendered acid, that this change is frequently overlooked. Few, I imagine, in the haste of pressing duties, fully realize how suddenly and seriously the milk is affected, by temporary causes, such as slight errors of diet, as by the use of green and unripe vegetables, and also most kinds of fresh fruit though ripe. The effect of these is to render the milk acid, which can be demonstrated by the simplest test.

The influence of diet upon the mother's milk should never be lost sight of when treating infants during the summer months. The immediate effects of acid milk upon the child are imperfect digestion and assimilation, giving rise to restlessness, wakefulness, etc., followed by vomiting and diarrhœa.

The inference is forced upon us, that attention to the regimen of the nurse, claims an important place in the management of the bowel affections of infancy. Manifestly, the articles of the mothers' diet should be restricted, to the exclusion of all acescents, but should be liberal in the quantity of plain nourishment. This leads me to add, that when the milk is deficient in quantity for the needs of the child, it is an error to attempt as is too often done, to increase the quantity by persistently overtaxing the stomach of the mother—with malt liquors—or other alcoholic stimulants, pottages, or even with solid food, to that degree that indigestion and anorexia are produced. Good milk is formed only when the general health is good, and the digestive organs perform their functions normally.

There are other qualities of the milk that not only fail to nourish the child, but produce derangements in its system, which lead to the most disastrous results. Such as the persistence of the elements peculiar to colostrum, or it may be depraved by the early return of menstruation, by the existence of pregnancy, by laborious occupations, by sexual excitement, by emotion and mental excitement, and by other causes which might be enumerated. Hence I conclude that it is proper to inquire into all causes which may impair the function of the mammary gland, ere we resort to the pencil and prescription paper.

No allusion to a substitute for the mother's milk, when that is unsuitable for the child, would be made, were it not a fact, that it is not always possible for parties to secure the offices of a hired wet nurse, and also for the other fact, that physicians permit, and sometimes advise, a resort to starch under one name or another, or to crackers and water, as the principal articles of food, upon the use of which by the infant, starvation, as a rule, should follow. The simple plan of allowing the milk of a well fed cow to stand for two hours, and then by taking the upper one-fourth with the cream properly diluted with warm water, and sweetened with *sugar of milk*, and given at the proper temperature always, will I believe answer the purpose well.

Suitable food is an essential in maintaining the health of the infant. Its development and growth require no less pure air and proper clothing. These constitute the tripod of life and health in early life. In many cases of sickness, all that is required is an adjustment of these essentials.

Were a criticism volunteered, upon the course too frequently followed when treating the diseases of infancy, it would be the too complete reliance placed on *medicine*, to the neglect of the hygienic conditions to which reference has been made. Is it not a fact that medical men are apt to fix the atttention exclusively upon the symptoms present, whether they be vomiting, diarrhœa, or abdominal pain, and at once set about removing these by medication, while they omit the weightier matters of the *law* of hygiene?

Nevertheless, medication may become necessary. When irritating substances have gained admission into the stomach and bowels, or when congestion or inflammation has set in, and the functions have been arrested or perverted, a resort to the means which experience has approved is imperative.

Vomiting, gastritis, diarrhœa and entero-colitis, are affections attributed to an elevated temperature, and these may be taken as a text for a review of the therapeutics on this occasion.

It will be understood I exclude those disorders of the alimentary canal, which are symptomatic of cerebral diseases or scarlatina, or are produced by other general diseases, and confine myself to the effects of unwholesome food and of abnormal secretions upon the mucous membrane, the irritability of which has been exalted and its tone depressed by high temperature, this atmosphere thus heated frequently introduces into the system, malaria, which aggravates all other symptoms.

Vomiting.—When offending material causes irritation of the stomach, the first indication, viz., its removal, is not infrequently effected spontaneously, by vomiting. In such cases the vomiting should not be considered a disease. It will be wise to withhold the ordinary nourishment for a time; for six, eight, or even for twelve hours, giving only the blandest fluids in small quantity, after which permit a return to the ordinary diet gradually, being assured, however, that the food does not contain offending material. Should the vomiting continue, a laxative may be administered, as castor oil; after its action, alkalies, lime water in milk, 5 to 50 Gms., one-fourth of which may be given every hour or two, and should the patient become restless, as is frequently the case, the following mixture may be found useful:

| | |
|---|---|
| Glycerine | 4 |
| Tr. opium camph | 4 |
| Cherry-laurel water | 8 |
| Syrup | 32 |

Mix. One teaspoonful every hour or two.

When the ejecta are intensely acid, the mistura cretæ may be substituted for the milk and lime water. If this be given for this condition it should be repeated at short intervals, every half hour in some cases. Failure in obtaining the desired results may be due, not to the remedy, but to the long intervals intervening between the doses. Strong coffee will also be found a good remedy, and in extreme cases is most efficient.

At a later period, and in a more severe form, attended with congestion or inflammation, characterized by febrile movement, tenderness over the epigastrium, dry tongue and great thirst, cooling bland fluids, alkalies, perfect quiet, and counter-irritation over the stomach are indicated. I have known a blister two inches square, applied for two hours to a child one year of age and over, effect complete relief. The admonition must not be omitted, that in early life it is dangerous to allow the blister to remain too long. When the skin is deeply reddened, remove the blister and apply a light poultice.

Diarrhœa is, many times, only an extension of the irritation starting in the stomach, or it may be a salutary effect of nature to remove offending material, which if retained, would lead to derangement of function and organic disease. You all well know how intractable and dangerous diarrhœa too frequently becomes, when the causes continue to act. It is important that nothing be omitted which will improve the hygienic condition of the patient. Attention should be directed to the quality and *quantity* of the food, and if fed artificially, especially to the *temperature* of the food. It is common for nurses to give the food at one time too hot and at another too cold. The air, the clothing to protect from sudden alterations of temperature, bathing, etc., must be under the supervision or at least under the special direction of the physician, who should also be particular to examine the condition of the gums. In brief, all causes which produce or aggravate irritation of the delicate mucous surface of the bowels should be removed.

When the dejections contain undigested substances as masses of casein, or are excessively acid, as they frequently are, and so acrid that the cutaneous surface is excoriated by their contact, a cathartic is indicated; oil in the first instance, calcined magnesia in the latter; relief will follow. To prevent a recurrence of the diarrhœa, follow the cathartic with pepsin (saccharated) one to three grains, every three or four hours, at the time of feeding. Also give the following:

| | |
|---|---|
| Bismuth sub-carb | 2 |
| Tr. opium camph | 4 |
| Syrup of ginger | 32 |

Mix. A teaspoonful as required.

Should the diarrhœa still continue, an astringent must be added, then substitute for the latter the following:

| | |
|---|---|
| Glycerine | 5 |
| Tr. kino | 5 |
| Tr. opium camph | 10 |
| Chalk mixture | 50 |

Mix. Teaspoonful as required.

The quantity of food must be limited, for if of good quality in excess, it will neutralize the good effects of medication.

The case will not continue long before symptoms of approaching exhaustion will be manifested. These should be detected early, for the prevention of exhaustion is easier than cure. For two reasons quinine is indicated at an early stage. First, it is reliable in arresting debility, and second, because malaria so frequently forms an important factor in the causation, quinine is *par excellence*, the remedy. When properly combined, any child will take it, if not with a relish at least without resistance. The following is suggested as an eligible formula:

| | | |
|---|---|---|
| Sulphate of quinine | | 40 |
| Tannic acid | | 20 |
| Powdered licorice root (Squibb's) | 4 | |
| Simple elixir, Syrup of tolu, } each | 16 | |

Mix. Teaspoonful every fourth or sixth hour.

Should the disease not yield, a new set of symptoms are liable to supervene, as increased frequency of the pulse, dry tongue, hot skin, increased restlessness, tenderness of abdomen; the discharges will be small in quantity and may contain mucus streaked with blood, indicative of inflammation. In this stage of the case I have to repeat it is important that acrid secretions be promptly removed from the alimentary canal. One teaspoonful of castor oil with one drop of laudanum will usually accomplish this. We must do more. An alterative will benefit these cases such as—

| | |
|---|---|
| Calomel .............................................. | 20 |
| Sugar of milk.........................................2 | |

Make ten powders.

Give one every three or four hours till the effect is perceptible in the secretions. At this stage the dejections are frequently characterized by a peculiarly offensive odor, as if produced by rapid decomposition. When this is the case I have found charcoal to act well. It may be given suspended in milk or cinnamon water, or syrup, or as in the following mixture:

| | |
|---|---|
| Bismuthic sub-carb ........................ ............ | 50 |
| Powdered charcoal.................................... 1 | |
| Tr. opium camph....................................... 2 | |
| Syrup of ginger.......................................32 | |

Mix. Teaspoonful every hour.

When the surface becomes hot and dry, a warm bath, followed by hot fomentations over the abdomen, on which a few drops of the ol. terebinthina have been sprinkled, sufficient to cause slight redness of the skin, will relax the surface and relieve the intestinal congestion. Should the evacuations occur frequently and contain lymph streaked with blood, then the following mixture should benefit:

| | |
|---|---|
| Plumbic subacet ..................................... | 10 |
| Morph. acetate........................................ | 016 |
| Acid acetic (crude)................................... | 30 |
| Syrup.............................................. 60 | |

Mix. Teaspoonful as required.

The case has now advanced to a stage when exhaustion follows rapidly. The nourishment should be varied, raw beef, essence of beef, or beef tea may be given; these will be acceptable to the stomach, and are easily assimilated. Stimulants, as brandy in water, will many times be useful in equalizing the circulation and in preventing a frequent complication, viz., spurious hydrocephalus. Tenesmus is a frequent and distressing symptom; an injection of 4 C. C. of mucilage of elm bark, containing one drop of laudanum, will afford relief.

In some cases the patients require an anodyne, when no form of opium is admissible. I have frequently prescribed the following, with satisfactory results :

| | | |
|---|---|---|
| Chloral hydrate | | 50 |
| Syrup | 32 | |

Mix. Teaspoonful as required.

It removes the irritability, quiets the restlessness which so frequently aggravates the debility, and many times will produce refreshing sleep.

Having arrested the violence of the symptoms, it is of the first importance to hasten the convalescence. For should slight symptoms only linger, the slightest causes may produce a relapse, more difficult to control than the first attack. Errors in diet, sudden variations of temperature and the like, are among the causes of relapse. The same vigilance is necessary during convalescence that was essential during the violence of the disease.

In some cases the circumstances of the parties will permit the best thing to be done, viz., to obtain the benefit of a change of air. Every gentleman present is familiar with the rapid improvement due to an exchange of city air for the atmosphere of the country. We have no mountain ranges within easy reach, that would give us an atmosphere best suited to these cases, but we have, what may be considered a great boon, the pure air on the lake. A trip to Mackinac, for those who can afford it, may be advised with great confidence. And for those who for any reason cannot take the trip, temporary but repeated sojourns on the lake will accomplish much. The hospital boat, in a large class of cases, affords the means essential to the successful management of the convalescence from cholera infantum, diarrhœa, etc.

This sanitary measure should be well patronized during the summer months. If one boat is not sufficient others should be employed, and at the public expense if necessary; the charge would not be burdensome.

Much benefit may be attained in cases where the best course is for any reason impracticable, by changing the patient from one room to another, even in the same house. Especially should the change be made to some upper room. The greater the elevation the better, for obvious reasons.

I have only to suggest, in conclusion, that heroic medication, when delicate structures are involved, may sometimes, with advantage, give place to lenitive measures, whose efficiency has stood the test of time and the experience of the profession. The best results are to be expected when suitable agents are accurately adapted to the pathological conditions present in any given case. I therefore close, as I began, by reiterating that the object of the paper has been to emphasize the importance of securing the best hygienic influences, both in preventing and removing the diseases of infancy in the summer months.

The baleful influences of a vitiated atmosphere upon the blood and tissues of animal life is patent to all in the community. That this influence constitutes an unknown quantity in the causation of the bowel affections of infancy and childhood, is undoubtedly true; still it is among the most potent for evil. If an absolutely pure atmosphere cannot be obtained in large cities, we should at least *strive* to attain the ideal, by avoiding and removing every source of contamination. To accomplish this, it is the duty of every good citizen to aid, by every legitimate means, in the accomplishment of this object. In this direction the exertions of our efficient Health officer, in improving the sanitary condition of the city, entitles him to the thanks of a long-suffering and afflicted community.

Those who for mercenary ends persist in poisoning the atmosphere, upon which all subsist, with deleterious gases from decomposing animal matter, thus causing and aggravating disease, in direct violation of the statute in such case made and provided, should be dealt with as other outlaws, in the last extremity, if necessary—by force.

---

THE Bulletin Printing Company, of Chicago, who do the press work for this Journal, can supply blanks properly ruled for prescription writing in accordance with the metric system; and Messrs. Buck & Rayner, retail druggists, of this city, are prepared to compound medicines written in the terms of the same system.

